# Associations between Stress, Anxiety, Depression and Sleep Quality among Healthcare Students

**DOI:** 10.3390/jcm12134340

**Published:** 2023-06-28

**Authors:** Monira Alwhaibi, Noha A Al Aloola

**Affiliations:** 1 Medication Safety Research Chair, College of Pharmacy, King Saud University, Riyadh 11149, Saudi Arabia; 2 Department of Clinical Pharmacy, College of Pharmacy, King Saud University, Riyadh 11149, Saudi Arabia

**Keywords:** sleep quality, perceived stress, anxiety, depression, healthcare students

## Abstract

Objectives: Stress, anxiety, and depression among students have many negative health consequences and may predispose students to poor sleep quality; therefore, this research aimed to investigate the perceived stress, anxiety, and depression and their relation to the level of sleep quality among healthcare students. Methods: A cross-sectional study using a validated survey was conducted among Saudi healthcare students from different regions during the period from 26 September 2022 to 30 October 2022. The Pittsburgh Sleep Quality Index (PSQI) was utilized to assess sleep quality. PSPP Statistical Analysis Software version 1.2.0 was used for all statistical analyses. Results: 701 respondents participated in this research; the response rate was 73.8%. About 60% of the study sample was female, and the average age was 20.9 years. 80.3% of students have poor sleep quality; the participants’ mean sleeping hours per night was 6.81 ± 1.88 h. About three-quarters of students (72.9%) perceive themselves as stressed. A significant positive correlation was observed between sleep quality and perceived stress (r-value = 0.363), anxiety (r-value = 0.387), and depression (r-value = 0.347). Poor sleep quality was more likely among those with stress than those without stress (AOR = 1.79; 95% CI 1.07, 2.99) and two times more likely among students with cases of anxiety than those without anxiety (AOR = 2.07; 95% CI 1.10, 3.94). Conclusions: Our study highlights the high prevalence of poor sleep quality, anxiety, depression, and stress among healthcare students in Saudi Arabia. Students’ reported stress, anxiety, and residence region were associated with poor sleep quality. These results imply the necessity of regular screening and appropriate intervention for sleep problems, stressors, and anxiety among healthcare students.

## 1. Introduction

Sleep quality is an individual’s satisfaction with all aspects of the sleep experience [1,2]. This concept includes several aspects, such as sleep efficiency, sleep onset latency, duration, and the number of awakenings after sleep onset [1,2]. Several published studies reported that poor sleep quality is highly prevalent among healthcare students. For example, a systematic review of 57 published studies that included 25,735 medical students has estimated a pooled prevalence of poor sleep quality of 52.7% using the Pittsburgh Sleep Quality Index (PSQI) [3]. A substantial body of evidence suggests that poor sleep quality is associated with adverse physical and mental problems [4,5,6]. Insufficient sleep appeared to be associated with obesity, cardiovascular morbidity, and risk of diabetes [4]. Also, poor sleep quality was associated with students’ academic performance, life satisfaction, anger, and fatigue [7,8,9]. Additionally, adults who do not obtain sufficient sleep also have a higher risk of developing stress, anxiety, and depression [5].

The relationship between stress, anxiety, depression, and poor sleep quality has been highlighted in several published studies among healthcare students [10,11,12,13,14,15]. A study among nursing students reported that poor sleep quality was associated with stress and symptoms of depression and anxiety. Mishra et al., in their study among 284 undergraduate medical students, found that students with stress had twofold odds of poor sleep quality compared to those without stress [13]. Another study found that higher perceived stress levels were significantly associated with poor sleep quality [12]. In a survey of 1125 college students between 17 and 24 years old, more than 60% of participants were classified as poor sleepers. According to the students, tension and stress significantly related to sleep disturbance, and they were responsible for 24% of the variance in the sleep quality score [14].

Although the association between stress and sleep quality seems well established by several studies, the prevalence of poor sleep quality among healthcare students and its relation to stress and mental health has not been sufficiently addressed in Saudi Arabia. Most of the studies in Saudi Arabia focused on medical students only [16,17,18,19] and not students from other health science programs such as pharmacy and nursing students. Therefore, this study intended to examine the prevalence of poor sleep quality; evaluate the association between perceived stress, anxiety, and depression and sleep quality among healthcare students in Saudi Arabia. We hypothesize that stress, anxiety, and depression are all related to poor sleep quality. Findings from this study are crucial for understanding what contributes to poor sleep quality in this academic population, bringing this issue to the attention of decision-makers, and aiding in developing successful interventions to improve sleep quality among healthcare students and help them cope with the pressures of academic learning.

## 2. Methods

### 2.1. Study Design and Sample

A cross-sectional study was carried out among healthcare students in Saudi Arabia using an online survey. The data were collected from 26 September 2022 to 30 October 2022. The study sample was composed of undergraduate healthcare students from different healthcare schools. The inclusion criteria include undergraduate students of all levels of health science bachelor programs (medicine, pharmacy, dentistry, and nursing, applied medical sciences) who consented to participate in this study. Students from non-health science programs were excluded from the study.

### 2.2. Ethical Considerations and Consent to Participate

The study was approved by the Research Center of the Medical College of King Saud University and its Ethical Committee (Protocol No. E-21-6192). The informed consent was given to the participants to describe the study goal and the participant’s freedom to withdraw at any time. No participant identifiers were used in this study to guarantee the privacy of the participant’s information.

### 2.3. Questionnaire Development and Validation

An anonymous online survey was written in English language and composed of four sections pertaining to sociodemographic data (age, gender, nationality, and region of residence); type of health science program and year of study; perceived physical health (excellent/very good, good, and fair/poor); and health (sleep quality, perceived stress, depression, and anxiety). A group of researchers (n = 3) reviewed the questionnaire for the content and ease of understanding of the questions to ensure content and face validity; researchers’ comments were taken into account. The questionnaire’s clarity and suitability for the intended sample were pilots tested among 22 participants who completed a self-administered survey. Minor adjustments were made in response to the pilot group’s feedback, and their responses have not been included in the final analysis of the sample.

### 2.4. Data Collection/Data Source

The questionnaire was hosted on a Google form; the survey link was distributed online via email. First, the informed consent form was displayed in our online survey on the first page before any research questions were asked. The study’s overall goal, the risks and benefits, the methods for protecting participants’ privacy, their rights, and a statement that the participation was entirely voluntary are all explained to participants in the consent form. Next, after the participants’ consent was obtained, students who agreed to participate were asked to complete the questionnaires.

### 2.5. Outcome Variable (Sleep Quality)

Sleep quality was evaluated using the Pittsburgh Sleep Quality Index (PSQI) [12]; the PSQI contains 19 self-reported questions. The PSQI is the most widely used assessment tool to evaluate subjective sleep quality during the previous month, which covers a broad range of indicators relevant to sleep quality [20]. It has seven components: subjective sleep quality, sleep latency, sleep duration, habitual sleep efficiency, sleep disturbances, use of sleeping medication, and daytime dysfunction. Global PSQI score is the sum of the 7 component scores, ranging from 0–21. A global score of 5 or under indicates “good sleep quality”, and a score greater than 5 indicates “poor sleep quality”.

### 2.6. Key Variables: Perceived Stress

Perceived stress was evaluated using the Perceived Stress Scale-14 (PSS), a method for assessing psychological stress widely [21,22]. PSS is a self-reported scale that is used to evaluate “the degree to which individuals perceive stressful situations in their life” [23]. Each item in this scale is rated on a 5-point Likert scale, with 0 indicating “never” and 4 indicating “very often” [24]. After reversing positive items’ scores (i.e., items 4, 5, 6, 7, 9, 10, and 13) and summing up all scores, the total scores range from 0 to 56. A score above 28 points was considered stressed, and 0–28 points were considered unstressed.

Mental health (anxiety and depression) was evaluated using the hospital anxiety and depression scale (HADS) [20]. It has been widely used among undergraduate healthcare students. It consists of 14 items, seven items to measure the anxiety subscale and seven items to measure the depression subscale. A total subscale score of 11 and over indicates a probable case of depression or anxiety, a score of 8–10 indicates borderline, and a score of 0–7 is considered normal.

### 2.7. Other Variables

Independent variables included sociodemographic data, type of health science program, year of study, and perceived physical health. The perceived physical health was evaluated by a single-item self-rated question: “How is your physical health in general?” The categories for the responses were (1) Excellent, (2) Very Good, (3) Good, (4) Fair, and (5) Poor [25].

### 2.8. Statistical Analysis

All data were analyzed using PSPP Statistical Analysis Software version 1.2.0 (GNU PSPP, Boston, MA, USA). In order to compare sleep quality groups, univariate and multivariate analyses were performed using Chi-square tests, independent groups *t*-tests, and regression analysis. Pearson correlation was conducted between the continuous scores of anxiety, depression, perceived stress, and sleep quality. Regression assumptions were evaluated first to ensure that assumptions were met. Then, a binary logistic regression analysis was performed to examine the associations between sleep quality and reported stress, anxiety, and depression after considering various confounders. The findings were presented as adjusted Odds ratios (AORs) with 95% confidence intervals (CIs), and the level of significance was (*p*-value < 0.05).

## 3. Results

### 3.1. Characteristics of the Study Sample

From a total of 950 students who received our online survey, 701 students participated in this study; the response rate was 73.8%. The average age of the participants was 20.9 years. The majority of participants were female (60.0%). Most of the students were from the middle region (53.1%). Table 1 displays the characteristics of the participants.

### 3.2. Prevalence of Perceived Stress, Anxiety, Depression, and Sleep Quality

Almost 73.0% of students were stressed (Table 2). Around 52.9% reported having cases of anxiety, and 32.2% of the participants had cases of depression. In 80.3% of healthcare students, poor sleep quality was prevalent. The subjects’ average nightly sleep duration was 6.81 ± 1.88 h. The subjects’ average perceived stress was (30.88 ± 7.09), sleep quality (8.30 ± 2.73), anxiety (10.59 ± 4.21), and depression (8.60 ± 3.90).

### 3.3. Correlation between Anxiety, Depression, Perceived Stress, and Sleep Quality

Table 3 displays the correlation between anxiety, depression, perceived stress, and sleep quality. There was a positive correlation between sleep quality and perceived stress (r-value = 0.363, *p* < 0.05), anxiety (r-value = 0.387, *p* < 0.05), and depression (r-value = 0.347, *p* < 0.05).

### 3.4. Sleep Quality

An association was found between poor sleep quality and stress (*p* < 0.001), anxiety (*p* < 0.001), and depression (*p* < 0.001) (Table 4). For example, poor sleep quality was significantly higher in healthcare students who reported stress than those without stress (89.6% versus 73.3%, *p*-value < 0.001) and cases of depression than those without depression (90.3% versus 78.2%, *p*-value < 0.001).

An association was found between poor sleep quality and region of residence (*p* = 0.044) and perceived physical health (*p* < 0.001) (Table 5). For example, a higher percentage of poor sleep quality was among healthcare students who perceived their physical health as poor than those with excellent health (87.5% versus 76.6%, *p*-value < 0.001). However, no association between sleep quality and age, gender, or type of healthcare program was found (*p*-value was greater than 0.05). Some reported reasons by students for having difficulty sleeping in our study include; stress, overthinking, studying, coffee consumption, and family-related issues.

### 3.5. Factors Associated with Sleep Quality from Adjusted Regression Analysis

An adjusted regression analysis was used to identify factors associated with sleep quality (Table 6). Participants who had stress were more likely to have poor sleep quality than those without stress (AOR = 1.79; 95% CI 1.07, 2.99). Students with anxiety were twice as likely as those without anxiety to have poor sleep quality (AOR = 2.07; 95% CI 1.10, 3.94). Less frequently did students from the north, east, and south have poor sleep quality than those from the western region.

## 4. Discussion

The current study investigated the relationship between perceived stress, anxiety, depression, and poor sleep quality among healthcare students in Saudi Arabia. Results showed a high prevalence of poor sleep quality among healthcare students. Also, our findings highlighted that perceived stress and anxiety are essential factors that were associated with the odds of poor sleep quality.

Our findings of a prevalence of poor sleep quality in almost 80% of participants are higher than the reported prevalence from previous studies in Saudi Arabia (30–76%) [16,17,18,19,26,27,28] and the global pooled prevalence (52.7%) using the same assessment scale (i.e., Pittsburgh Sleep Quality Index) [3]. The variation in the prevalence of poor sleep quality reported in previous studies could be due to the difference in the study population or the different assessment methods used to measure sleep quality. Some reported reasons by students for having difficulty sleeping in our study include; stress, overthinking, studying, coffee consumption, and family-related issues. The availability and use of stimulants (like coffee) are related to changes in sleep patterns, an essential modifiable lifestyle risk factor for students. Caffeine is the most popular psychoactive substance used globally [29,30]. According to a recent systematic review of epidemiologic research and randomized clinical trials, caffeine use has been found to harm both subjective and objective sleep quality [29]. According to this review, caffeine was associated with perceived sleep quality, decreased total sleep time and efficiency, and prolonged sleep latency.

The results of this study highlighted a high prevalence of stress, anxiety, and depression among healthcare students. Anxiety was reported by half of students, stress affected nearly three-quarters, and depression affected one-third of participants. Our findings, which indicate a higher prevalence of stress, are consistent with previous research among Saudi medical students [17,31,32] but greater than what was reported globally (31.0–64.0%) [33,34]. Participants in the study reported high levels of anxiety and depression. The depression rate is consistent with the national and international rates among medical students [16,35,36], and the anxiety level was comparable to the documented prevalence in earlier studies among medical students worldwide and in Saudi Arabia [16,35,37,38].

This research found significant associations between perceived stress and anxiety with poor sleep quality. Perceived stress relation with poor sleep quality result aligns with the earlier studies that have underscored this meaningful relationship [16,17,26,35,39]. Another significant finding in this research was the association between anxiety and poor sleep quality. Those with anxiety cases were twofold to have poor sleep quality. In fact, anxiety has been previously reported to be linked to poor sleep quality in Saudi medical students [16,17,26]. Although those findings have been reported in earlier research, this study added to the existing literature the focus on this relationship among students in different healthcare fields, not only medical students. This study’s findings emphasize the need to offer healthcare students stress management interventions and coping techniques. Evidence suggests a connection between adults who stutter and sleep quality, stress, and anxiety [40]. Stuttering is a condition of the speech motor system. According to research, stuttering is linked to social anxiety, and lack of sleep is linked to increased anxiety levels. As a result, the degree of stuttering is correlated with the severity of sleep disturbances.

Age, gender, residence region, and the type of health science program were all examined in this study as potential influences on the quality of sleep. The residence region was associated with sleep quality, whereas age, gender, and sleep quality were not shown to be associated in our study [18]. These findings contradict past research, which showed that women experienced much higher levels of poor sleep quality than men [41]. The gender disparities in the prevalence of sleep quality exist in the univariate analysis but not in the adjusted analysis. Gender disparities were anticipated due to the differences in sociocultural factors such as income and biological factors such as sexual hormones; however, it was not statistically significant in our study. Additionally, the adjusted regression analysis revealed no significant change in sleep quality across several health science programs.

### 4.1. Study Implications

The current study has several potential applications. First, the increasing prevalence of poor sleep quality and its link to stress and anxiety underscores the need to enhance mental health services, which will ultimately enhance their sleep quality and improve health-related quality of life (HRQoL). Studies have demonstrated a relationship between the aspects of sleep quality and good physical and mental health, and good HRQoL [42,43,44,45,46]. For example, a recent study revealed that all of the distinct correlations between anxiety, depression, stress, and HRQoL were mediated by overall sleep quality. These preliminary results imply that medical students’ well-being is related to the quality of their sleep and that it may be advantageous to address sleep problems in this academic population [42].

Additionally, considering that improving sleep quality is associated with enhanced physical and mental health [47], future research should evaluate healthcare students’ sleep knowledge and provide them with sleep education. This training must cover subjects including sleep and circadian science, sleep hygiene, and the clinical evaluation and treatment of sleep disturbances and disorders [48]. Some evidence of the effectiveness of sleep education programs in enhancing sleep hygiene knowledge, sleep hygiene behavior, and/or sleep quality when compared to traditional techniques has been documented by a comprehensive review of four interventional trials [49].

### 4.2. Strength/Limitations

This study evaluated sleep quality and the association between stress, anxiety, and depression and poor sleep quality in different healthcare schools in Saudi Arabia. In contrast, previous studies focused only on medical students. However, it is important to take into account some of this study’s limitations. Due to the study design’s cross-sectional nature, we cannot evaluate the causal relationship. Also, other confounders, such as the use of social media, family support, physical activity, and academic performance, needed to be measured and adjusted. Also, this study did not explore the other reasons that are associated with poor sleep quality, which could be either physiological (e.g., body mass index), environmental (e.g., television/device use), or a combination [1]; thus, future research should address this. Additionally, we cannot rule out recall bias since the data were self-reported.

## 5. Conclusions

Our study highlights the high prevalence of poor sleep quality, anxiety, depression, and stress among healthcare students in Saudi Arabia. Students’ reported stress, anxiety, and residence region were associated with poor sleep quality. These results imply the necessity of regular screening and appropriate intervention for sleep problems, stressors, and anxiety among healthcare students to enable prompt assistance and support from social work educators to consider including sleep hygiene education and offer counseling and stress management interventions to help healthcare students obtain better sleep and cope with stressors in academic education.

## Figures and Tables

**Table 1:** Baseline Characteristics of the Study Sample (n = 701).

Variables		N	%
**Total**		701	100
**Age Mean (SD)**		20.94 ± 1.92	
**Gender**	Male	285	40.7
	Female	416	59.3
**Residence Region**	North	76	10.8
	East	85	12.1
	Middle	372	53.1
	South	62	8.8
	West	106	15.1
**Program**	Medicine	205	29.2
	Dentistry	71	10.1
	Pharmacy	178	25.4
	Nursing	99	14.1
	Applied Medical Science	148	21.1
**Perceived Health**	Excellent	154	22.0
	Very Good	222	31.7
	Good	237	33.8
	Fair or Poor	88	12.6

**Table 2:** Prevalence of stress, anxiety, depression, and sleep quality.

		N	%
**Total**		701	100
**Perceived Stress**	Stressed	509	72.6
	Not Stressed	192	27.4
**Anxiety**	Normal	168	24
	Borderline	162	23.1
	Case	371	52.9
**Depression**	Normal	252	36
	Borderline	223	31.8
	Case	226	32.2
**Sleep Quality**	Normal	105	15
	Poor	563	80.3
	Missing	33	4.7

**Table 3:** Correlation between Pittsburgh Sleep Quality Index and other parameters.

Variables	Pittsburgh Sleep Quality Index	
	**r**	***p* Value**
**Perceived stress**	0.363	<0.05
**Anxiety**	0.387	<0.05
**Depression**	0.347	<0.05

**Table 4:** Relationship between stress, anxiety, depression, and sleep quality (n = 701).

		Normal Sleep Quality		Poor Sleep Quality			
		N	%	N	%	Chi-Square Value	*p*-Value
Total		105	15	563	80.3		
**Stress**	Stressed	53	10.4%	456	89.6%	28.78	<0.001
	Not stressed	51	26.6%	141	73.4%		
**Anxiety**	Normal	48	28.6%	120	71.4%	33.27	<0.001
	Borderline	19	11.7%	143	88.3%		
	Case	37	10.0%	334	90.0%		
**Depression**	Normal	55	21.8%	197	78.2%	15.71	<0.001
	Borderline	27	12.1%	196	87.9%		
	Case	22	9.7%	204	90.3%		

*p*-value presents differences in stress from chi-square tests.

**Table 5:** Association between sleep quality and other study variables.

		Normal Sleep Quality		Poor Sleep Quality			
		N	%	N	%	Chi-Square Value	*p*-Value
Total		105	15	563	80.3		
**Age Mean (SD)**		21.05 ± 1.81		20.9 ± 1.96			0.372
**Gender**	Male	46	16.1%	239	83.9%	0.65	0.45
	Female	58	13.9%	358	86.1%		
**Residence Region**	North	15	19.7%	61	80.3%	9.8	0.044
	East	19	22.4%	66	77.6%		
	Middle	48	12.9%	324	87.1%		
	South	12	19.4%	50	80.6%		
	West	10	9.4%	96	90.6%		
**Program**	Medicine	33	16.1%	172	83.9%	3.62	0.46
	Dentistry	8	11.3%	63	88.7%		
	Pharmacy	27	15.2%	151	84.8%		
	Nursing	10	10.1%	89	89.9%		
	Applied Medical Science	26	17.6%	122	82.4%		
**Perceived Health**	Excellent	36	23.4%	118	76.6%	16.3	<0.001
	Very Good	36	16.2%	186	83.8%		
	Good	21	8.9%	216	91.1%		
	Fair or Poor	11	12.5%	77	87.5%		

*p*-value presents differences in stress from chi-square tests and *t*-test.

**Table 6:** Statistics from logistic regression of the factors associated with sleep quality.

		Poor Sleep Quality		
Variables		AOR	95% CI	*p*-Value
**Stress**	Stress	1.79	(1.07, 2.99)	0.026 *
	No Stress (Ref.)			
**Anxiety**	Borderline	2.08	(1.10, 3.94)	0.024 *
	Case	2.07	(1.14, 3.75)	0.017 *
	Normal **(Ref.)**			
**Depression**	Borderline	1.29	(0.72, 2.29)	0.387
	Case	1.33	(0.71, 2.51)	0.377
	Normal **(Ref.)**			
**Age**		0.95	(0.85, 1.08)	0.449
**Gender**	Female	0.86	(0.54, 1.39)	0.547
	Male **(Ref.)**			
**Residence Region**	North	0.42	(0.17, 1.05)	0.063
	East	0.36	(0.15, 0.89)	0.026 *
	Middle	0.71	(0.32, 1.53)	0.378
	South	0.34	(0.13, 0.90)	0.029
	West **(Ref.)**			
**Program**	Medicine	1.07	(0.58, 1.99)	0.820
	Dentistry	1.82	(0.73, 4.54)	0.200
	Nursing	1.56	(0.66, 3.69)	0.315
	Applied Medical Science	0.88	(0.47, 1.66)	0.702
	Pharmacy **(Ref.)**			
**Perceived Health**	Excellent	0.67	(0.30, 1.49)	0.328
	Very Good	0.90	(0.42, 1.92)	0.776
	Good	1.70	(0.76, 3.80)	0.197
	Fair or poor **(Ref.)**			

* asterisk represents significant findings. AOR: adjusted Odds ratio; CI: confidence interval; Ref: Reference Group.

## Data Availability

The corresponding author will provide the datasets used and analyzed during the current work upon reasonable request.
